# The Middlesex Hospital: Reports of the Medical, Surgical, and Pathological Registrars for the Year 1889

**Published:** 1892-06

**Authors:** 


*The Middlesex Hospital: Reports of the Medical, Surgical, and Pathological Registrars for the Year* 1889. Pp. 332. London: H. K. LEWIS. 1891.

This volume gives, as usual, a complete record of the work of the hospital during the year, and thus contains a number of valuable clinical and pathological observations. We note that in the report of the surgical registrar the tables of diseases and injuries have been this year arranged in accordance with the nomenclature of diseases recommended by the Royal College of Physicians, and the cases further tabulated according to the results of treatment. In each case where the uterus or its appendages were removed, an account of the pathological changes found in the organs removed is given—an essential adjunct to the clinical record of the case. In his pathological report, Dr. Sidney Martin has given an appendix to the cases of tuberculosis, "classifying those diseases which caused death and in which active or 'healed' tubercle was present."

